# Activation of the LINC00242/miR-141/FOXC1 axis underpins the development of gastric cancer

**DOI:** 10.1186/s12935-020-01369-7

**Published:** 2020-06-24

**Authors:** Xiongdong Zhong, Xianchang Yu, Xiaoyan Wen, Lei Chen, Ni Gu

**Affiliations:** Department of General Surgery, Zhuhai People’s Hospital (Zhuhai hospital affiliated with Jinan University), No.79 Kangning Road, Xiangzhou District, Zhuhai, 519000 Guangdong China

**Keywords:** Gastric cancer, LINC00242, MicroRNA-141, FOXC1

## Abstract

**Background:**

Long non-coding RNAs (LncRNAs) are a class of newly identified transcripts recognized as critical governors of gene expression during human carcinogenesis, whereas their tumor-suppressive or tumor-promoting effects on gastric cancer (GC) are required for further investigation. In the study, we identify the expression pattern of a novel lncRNA LINC00242 in GC and its possible permissive role in the development of GC.

**Methods:**

The study included 68 pairs of GC and adjacent normal gastric tissue samples. The viability, migration, and invasion of cultured human GC cells HGC27 were evaluated by CCK-8 and Transwell chamber assays. In vitro tube formation of human brain microvascular endothelial cells (HBMVECs) in HGC27 cell coculture was detected. The regulatory network of LINC00242/miR-141/FOXC1 was verified using dual luciferase reporter gene assay and RNA immunoprecipitation (RIP) assay. Subcutaneous xenografts of HGC27 cells were performed in nude mice.

**Results:**

LINC00242 was highly expressed in GC tissues and cells and contributed to poor prognosis. LINC00242 knockdown inhibited HGC27 cell viability, migration and invasion, and tube formation of HBMVECs. LINC00242 interacted with miR-141 and positively regulated FOXC1, a target gene of miR-141. LINC00242 knockdown was partially lost in HGC27 cells upon miR-141 inhibition or FOXC1 overexpression. The tumor-promoting effect of LINC00242 on GC was demonstrated in nude mice.

**Conclusion:**

Taken together, the present study demonstrates the oncogenic role of the LINC00242/miR-141/FOXC1 axis in GC, highlighting a theoretical basis for GC treatment.

## Background

According to Global Cancer Statistics 2018, gastric cancer (GC) ranks the 5th most frequently occurring cancer, accounting for 5.7% in both sexes on a global scale, and responsible for 8.2% of total cancer-related deaths [1]. It has been reported that there are several common therapeutic schedules for GC, such as surgery, radiotherapy, chemotherapy, and targeted therapy [2]. Although there were great advancements in the treatment for GC, the survival rate for advanced GC remains poor [3]. Accumulating evidence has revealed that GC is often accompanied by angiogenesis [4]. Angiogenesis is a complicated process to form new blood vessels from pre-existing vessels, and disruption of tumor angiogenesis could affect tumor growth and metastasis [5]. Thus, identification of the critical molecules involved in angiogenesis may provide breakthroughs for GC treatment.

LncRNAs exert their functions at the translational, transcriptional, and posttranscriptional level and are implicated in the occurrence and development of many human cancers, including GC [6]. Moreover, it’s interesting to note that multiple lncRNA may be playing an important role in angiogenesis in GC [7], while roles of lncRNA LINC00242 in GC remains unclear. microRNAs (miRNAs), a group of small non-coding RNA molecules, serve as regulators of inhibition of mRNA translation or reduction of mRNA stability by binding to the 3′-untranslated region (3′-UTR) of target mRNA [8]. miR-141, belongs to the miR-200 family, is poorly expressed in GC and implicated in the GC development by regulating cell proliferation, invasion, and metastasis [9]. Transcriptome analysis demonstrates the presence of miRNA-lncRNA-mRNA interaction in the malignant transformation process of GC initiation [10]. For example, Zhou et al. have validated that lncRNA H19 competed with miR-141 exerts effects on GC [11]. A bioinformatics website predicts that forkhead box (Fox) C1 is a potential target gene of miR-141. FOXC1 is also proved to be participated in GC [12]. FOXC1, a member of the FOX family, is essential for cell proliferation, migration, invasion, as well as vascular formation and maturation [13, 14]. However, their specific mechanism in angiogenesis of GC remains largely unknown. In the present study, the role of the newly discovered lncRNA LINC00242 in the cell invasion, metastasis, and angiogenesis of GC and its possible mechanisms is explored, which may help to provide a novel direction for GC treatment.

## Materials and methods

### Ethics statement

The Ethics Committee of Zhuhai People’s Hospital has approved the study protocol. We receive informed written consents from all participants and do our best to minimize the number and suffering of animals.

### Tissue specimen collection

Tumor tissues and their matched normal tissues adjacent to tumor were surgically resected from 68 GC patients who were admitted into the Zhuhai People’s Hospital from January 2013 to December 2015. None of the included patients received chemotherapy or radiotherapy. The 68 patients were seen for follow-up visits for 5–60 months and 9 patients were lost to follow-up.

### Cell harvest and transient transfection

Human GC cell lines, SGC7901, BGC823, MKN45, HGC27, and a normal gastric epithelial cell line GES-1 (ATCC, USA) were harvested in DMEM medium supplemented with 10% fetal bovine serum (FBS) (Gibco, USA), 100 U/mL penicillin, and 100 mg/mL streptomycin (5% CO_2_, 37 °C). Cultured HGC27 cells were treated with miR-141 mimic (#4464066), miR-141 inhibitor (#4464084), three anti-LINC00242 siRNA constructs (si-LINC00242-1, si-LINC00242-2, and si-LINC00242-3), expression vectors containing the FOXC1 gene (oe-FOXC1), and their negative control (NC) [mimic-NC, inhibitor-NC, scramble siRNA (si-NC), and empty vector (oe-NC)] (all purchased from Thermo Fisher Scientific, USA) alone or in combination as required by using lipofectamin 2000 reagents (11668-019, Invitrogen, USA) for 48 h according to the manufacturer’s instructions. The sense of si-LINC00242: 5′-UAUCUCCAAGGCAUGGAGC-3′; the anti-sense of si-LINC00242: 5′-GCUGGAUGCCUUGGAGAUA-3′. The sense of si-NC: 5′-UAUGGCUCGAACUACGAGC-3′; the anti-sense of si-NC: 5′-GCUCGUAGUUCGAGCCAUA-3′.

### RNA isolation and quantification

Total RNA was extracted from GC tissues or HGC27 cells by using the TRIZOL kit (Invitrogen, USA) following the manufacturer’s instructions. Then, miRNA was reversely transcribed to cDNA with reference to the manual of TaqMan MicroRNA Assays Reverse Transcription prime whereas mRNA was reversely transcribed to cDNA according to instructions of EasyScript First-Strand cDNA Synthesis SuperMix (AE301-02, TransGen Biotech, Beijing, China). Reverse transcription quantitative polymerase chain reaction (RT-qPCR) was executed following the steps described in the instructions of SYBR^®^Premix Ex TaqTM II kit (TaKaRa, Dalian, Liaoning, China) by using ABI 7500 instrument (Applied Biosystems, Foster City, CA, USA). The expression of miR-141 was relative to U6 while that of the other genes was obtained with GAPDH as a loading control. The synthesis of primer sequences was committed to Shanghai Majorbio Co., Ltd., (Shanghai, China) (Table 1). Finally, the fold changes were calculated by means of relative quantification (2^−ΔΔCt^ method).

**Table 1:** Primer sequences used for RT-qPCR

Target	Primer sequences (5′–3′)
LINC00242	F: TTCAGGCGCTGTCTGTTCTT
	R: TGCGAATCGATGGGGATCAG
FOXC1	F: ACTCGGTGCGGGAGATGTT
	R: CCTTGATGGGTTCCTTTAGC
miR-141	F: ACACTCCAGCTGGGCATCTTCCAG
	R: CTCAACTGGTGTCGTGGAGTCGGC
U6	F: CTCGCTTCGGCAGCACA
	R: AACGCTTCACGAATTTGCGT
GAPDH	F: TGAAGGTCGGAGTCAACGG
	R: CTGGAAGATGGTGATGGGATT

*F* forward, *R* reverse

### Western blot analysis

GC tissue homogenates or HGC27 cells were lysed by Radio Immunoprecipitation Assay lysis (P0013B, Beyotime Institute of Biotechnology Co., Ltd., Shanghai, China) supplemented with 1 mM phenylmethylsulfonyl fluoride. The protein sample was separated by the method of the SDS-PAGE and transferred onto a polyvinylidene fluoride membrane. The membrane was probed with diluted primary antibodies (Abcam Inc., Cambridge, UK) [N-cadherin (1:1000, ab18203), Vimentin (1:1000, ab92547), MMP-2 (1:1000, ab37150), MMP-9 (1:1000, ab73734), VEGF (1:1000, ab53465), CD31 (1:1000, ab134168), GAPDH (1:1000, ab181602) overnight at 4 °C. Immunoblots were visualized using goat anti-rabbit immunoglobulin G (IgG) (1:20000, ab6721, Abcam Inc.) and the enhanced chemiluminescence reagent. The gray values were assessed by means of Image J software (National Institutes of Health, Bethesda, Maryland).

### Cell viability assays

Cells were seeded into 96-well plates (1 × 10^5^ cells/ml) and then allowed to react with 10 μl CCK-8 (35000, AAT Bioquest, Mercury Drive, Sunnyvale, CA, USA) every other day at 37 °C. Four hours later, the medium was replaced by 150 μL Dimethyl Sulfoxide (DMSO, Sigma-Aldrich, USA) in each well to dissolve the formazan crystals. Absorbance was read at 450 nm using a Microplate reader (DNM-9602G; Aolu Biotech, Shanghai, China).

### Dual luciferase reporter gene assay

Artificially synthesized FOXC1 3′untranslated region (UTR) fragments containing putative miR-141 binding sites were inserted into the pmirGLO (Promega, USA) (named FOXC1-WT). A complementary sequence mutation site of putative miR-141 binding sites was designed on FOXC1 3′UTR and likewise inserted into the pmirGLO (named FOXC1-MUT). HEK293T cells (Shanghai Beinuo Biotech Ltd., Shanghai, China) were seeded into 6-well plates in triplicate and co-transfected with well-designed pmirGLO-based reporter plasmids and miR-141 mimic using the dual-luciferase reporter assay system according to the manufacturer’s instructions (D0010, Beijing Solarbio Science & Technology Co., Ltd, China). The luminescence of the firefly luciferase was normalized to the luminescence of the renilla luciferase.

### Transwell invasion and migration assays

Transwell invasion assays were performed to detect cell invasion. The 200 μL serum-free medium was added with 200 μL Matrigel. Cells were prepared into cell suspension using medium containing 20% FBS. Each well in the apical chamber-coated with Matrigel was added with 200 μL cell suspension, and the basolateral chamber was added with 800 μL conditioned medium containing 20% FBS. Transwell chambers were maintained at 37 °C. Twenty hours later, the cells in the basolateral chamber were stained with 0.1% crystal violet. Cells were observed, photographed, and counted under the inverted microscope. Transwell migration assays were performed in the absence of Matrigel, lasting for 16 h.

### Microtube-forming assays

Human brain microvascular endothelial cells (HBMVECs) were seeded into a Matrigel-coated 96-well plate with 2.5 × 10^4^ cells for each well. Once adhered to the wall, HBMVECs were incubated with GC cell supernatant for 4–6 h.

### Fluorescence in situ hybridization (FISH)

We used lncRNA subcellular localization data which are available at lncatlas.crg.eu. to predict subcellular localization of LINC00242. Then, FISH was performed to further examine the subcellular localization of LINC00242 using Ribo™ lncRNA FISH Probe Mix (Red) (Ribo Biotech, Guangzhou, China) as per the manufacturers’ instructions. In detail, cells were mounted onto slides and fixed in 4% formaldehyde. Slides were pretreated by protease K (2 μg/mL), glycine and acetic anhydride, followed by prehybridization for 1 h at 42 °C. Hybridisation was performed overnight at 42 °C using probes (250 μL, 300 ng/mL) against LINC00242. Finally, slides were stained with phosphate-buffered saline with Tween (PBST)-diluted 4′,6-diamidino-2-phenyl indole (DAPI). Images were acquired with the fluorescence microscope (Olympus, Japan), with five random fields acquired from each slide.

### RNA immunoprecipitation (RIP)

Cells were lysed in RIP Lysis Buffer at 4 °C for 30 min. Cell extracts were incubated with protein A/G Sepharose beads conjugated with antibodies against Ago2 (P10502500, Otwo Biotech, Shenzhen, China) or normal mouse IgG. Immunoprecipitated RNA and total RNA from the whole cell lysates (input controls) were extracted for qRT-PCR analysis.

### Tumorigenicity assay in nude mice

A total of 14 BALB/c nude mice (aged 4 weeks old and weighing 18–22 g) were purchased from Hunan SLAC Laboratory Animal Co., Ltd. (Changsha, China). Cell suspension (5 × 10^7^/ml) made from cells stably expressing shRNA targeting LINC00242 or scramble shRNA was subcutaneously injected into the nude mice to establish models of subcutaneous transplantation of GC in nude mice. Tumor growth was recorded every 3 days. Fifteen days later, mice bearing subcutaneous tumors were euthanized by cervical dislocation.

### Immunohistochemistry

Paraffin-embedded sections (5 μm) were dewaxed and hydrated and immunostained with the primary antibody, anti-VEGF (1:1000, ab53465, Abcam) or anti-CD31 (1:1000, ab134168, Abcam) at 4 °C overnight. Next, the sections were incubated with goat anti-rabbit Immunoglobulin G (IgG) (ab6721, 1:1000, Abcam) secondary antibody. Subsequently, the sections were developed by the diaminobenzidine (DAB) (ST033, Guangzhou Weijia Technology Co., Ltd., Guangzhou, China) and counterstained with hematoxylin (PT001, Shanghai Bogoo Biotechnology Co., Ltd., Shanghai, China) for 1 min. Positive-stains were photographed under the microscope. Five high power fields were randomly selected in each slice (100 cells/field).

### Statistical analysis

Results are presented as mean ± standard deviation (s.d.) of three technical replicates. Student’s *t* test (paired or unpaired), one-way analysis of variance (ANOVA) followed by Tukey’s test, and repeated measurements ANOVA with Bonferroni corrections were used for statistical comparisons as required. A value of *p *< 0.05 indicated statistical significance using the SPSS 21.0 software (IBM Corp. Armonk, NY, USA).

## Results

### LINC00242 was upregulated in GC tissues and cells

**Fig. 1:**
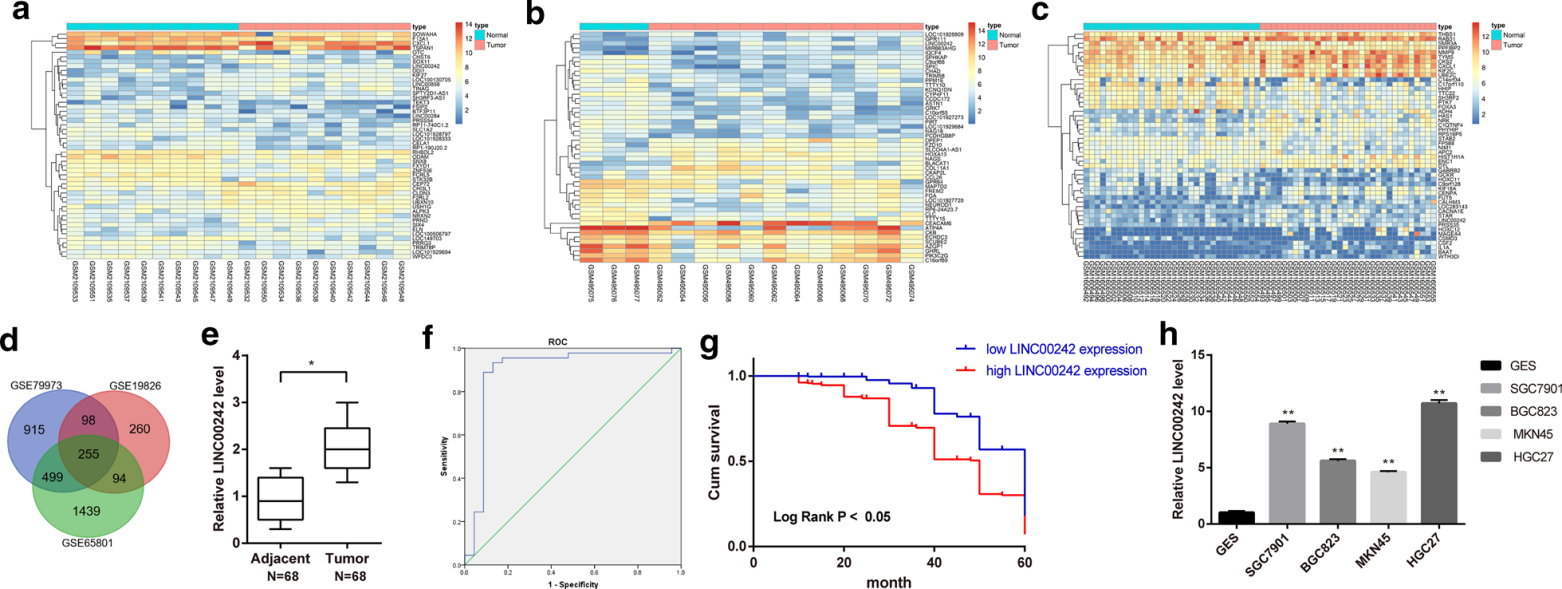
Expression pattern of LINC00242 in GC tissues and cells. a–c, Heat maps depicting the DEGs obtained from the GC-related datasets GSE79973, GSE19826 and GSE65801. The abscissa represents the sample number, and the ordinate represents the differentially expressed gene; the left dendrogram represents gene expression cluster; each rectangle corresponds to a sample expression value; the histogram at the upper right refers to color gradation. **d** Venn analysis of DEGs in GC microarray, three circles in the figure respectively represent the DEGs from the three datasets, and the middle part represents the overlaps. **e** The expression of LINC00242 in 68 pairs of GC and adjacent normal gastric tissues was determined by RT-qPCR. **f** The area under the ROC curve according to LINC00242 expression. The threshold of LINC00242 expression was 0.803. **g** The Kaplan–Meier method was used to plot GC patient survival according to LINC00242 expression. **f** The expression of LINC00242 in GES, SGC7901, BGC823, MKN45 and HGC27 cell lines was determined by RT-qPCR. Results are presented as mean ± S.D. of three technical replicates. *(compared with adjacent normal gastric tissues) indicates *p *< 0.05 by paired *t*-test. ** (compared with GES cells) indicates *p *< 0.01 by one-way ANOVA followed by Tukey’s test

We firstly obtained differential expression genes (DEGs) between GC tissues and normal tissues by analyzing raw data of GC-related expression datasets GSE79973, GSE19826 and GSE65801 that deposited in the Gene Expression Omnibus (GEO). Differential expression analysis was performed by using Bioconductor Project’s (http://www.bioconductor.org) open-source software in R statistical programming language. Based on p value (p < 0.05) and fold change (twofold as a cutoff), 1767, 707 and 2287 DEGs stood out from these three datasets, respectively. A heat map illustrating the top 50 DEGs is shown in Fig. 1a–c. Subsequent results (Fig. 1d) displayed 255 overlapping genes with significant difference in the three datasets, among which, there was one lncRNA, LINC00242, which was highly expressed in GC in the three datasets. Next, we collected clinical GC tissue and adjacent normal gastric tissue samples to detect the expression of LINC00242, and found that LINC00242 was abundantly expressed in GC tissues (Fig. 1e). The threshold of LINC00242 expression was obtained from the area under the receiver operating characteristic (ROC) curve. As shown in Fig. 1f, the area under the ROC curve is 0.894, with cut-off value of 0.803. Gastric cancer patients with LINC00242 expression of more than 0.803 were categorized into high-expressed group and those with LINC00242 expression of less than 0.803 were categorized into low-expressed group. Moreover, the results depicted in Table 2 showed that the expression of LINC00242 in GC was associated with the degree of tumor differentiation, lymph node metastasis and TNM stage (*p* < 0.05), but not with the gender, age and tumor size (*p *> 0.05). In addition, the overall 5-year survival rate of GC patients with high LINC00242 expression was lower than that of GC patients with low LINC00242 expression (Fig. 1g). These results suggested that the high expression of LINC00242 in cancer tissues can predict the adverse prognosis of patients with GC. Subsequently, RT-qPCR analysis revealed that LINC00242 expression was upregulated in GC cell lines of SGC7901, BGC823, MKN45, HGC27 compared with normal gastric epithelial cell line GES (*p* < 0.05) (Fig. 1h), with the HGC27 cell lines showing the highest LINC00242 expression. Therefore, HGC27 cell lines were selected for the following experiments.

**Table 2:** Correlation of LINC00242 expression with the clinical characteristics of GC patients

Clinical characteristics	Case	LINC00242 expression		χ^2^	*p* value
		high (n)	low (n)		
Gender				0.515	0.473
Male	39	21	18		
Female	29	19	10		
Age				0.020	0.451
≤ 60	48	29	19		
> 60	20	11	9		
Lymph node metastasis				6.773	0.009*
No	22	8	14		
Yes	46	32	14		
TNM stage				10.105	0.018*
I	13	4	9		
II	11	4	7		
III	20	15	5		
IV	24	17	7		
Tumor differentiation				4.404	0.036*
High and medium	23	9	14		
Low and none	45	31	14		
Tumor size				1.060	0.303
≤ 4 cm	35	18	17		
> 4 cm	33	22	11		

Data were analyzed using Chi square test (*p < 0.05)

### LINC00242 knockdown represses GC cell migration, invasion, and in vitro angiogenesis

**Fig. 2:**
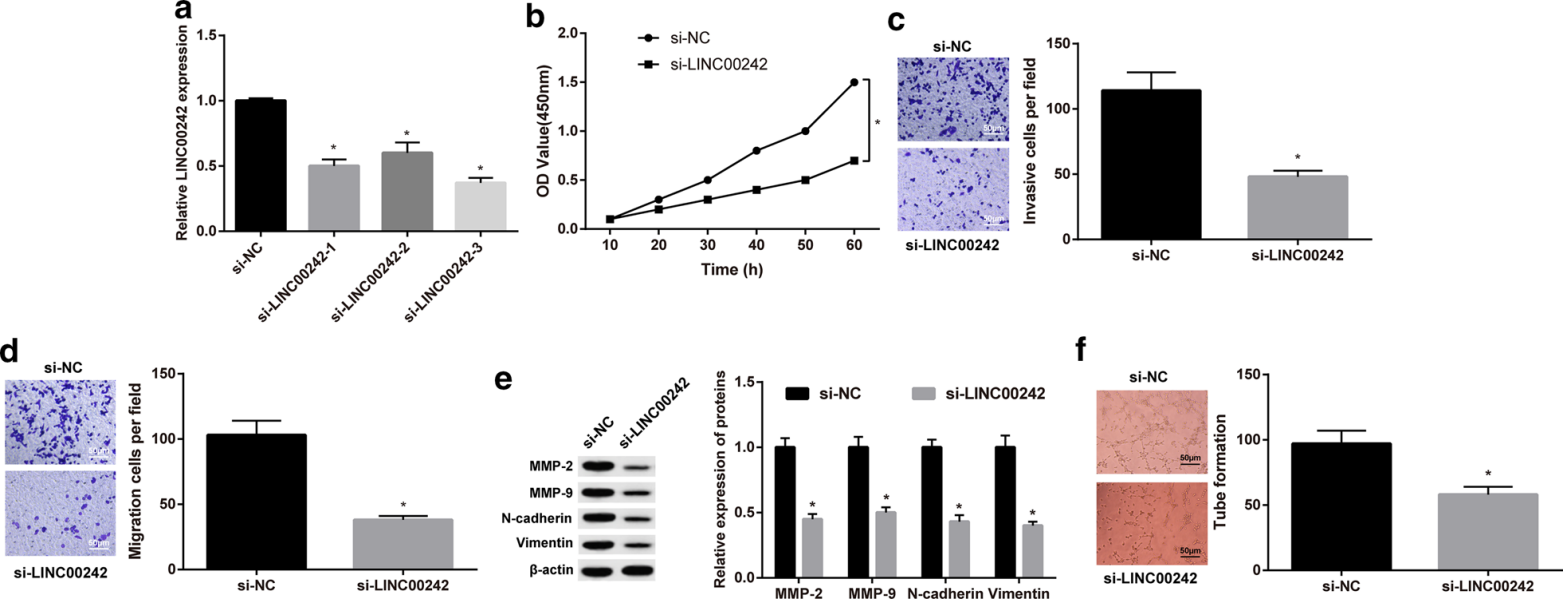
LINC00242 knockdown represses GC cell migration, invasion, and in vitro angiogenesis. **a** The expression of LINC00242 in HGC27 cells upon LINC00242 knockdown was determined by RT-qPCR. **b** HGC27 cell viability was measured by CCK-8 assay. **c** Representative view of HGC27 cells with LINC00242 knockdown invading from Matrigel-coated chambers into lower ones and statistics of invading cells. **d** Representative view of HGC27 cells with LINC00242 knockdown migrating from upper transwell chambers into lower ones and statistics of migrating cells. **e** Representative Western blots of N-cadherin, Vimentin, MMP-2, MMP-9 and their quantitation analyses in HGC27 cells upon LINC00242 knockdown. **f***In vitro* tube formation of HBMVECs in HGC27 cells upon LINC00242 knockdown. Results are presented as mean ± S.D. of three technical replicates. *indicates *p *< 0.05 compared with si-NC by unpaired *t*-test (panel **a, c–f**) or repeated measurements ANOVA with Bonferroni corrections (panel **b**)

In this part, we set out to study the effect of LINC00242 on the biological functions of GC cells. Firstly, we knock downed LINC00242 in HGC27 cells. To eliminate the influence of off-target, we set up three anti-LINC00242 constructs, si-LINC00242-1, si-LINC00242-2 and si-LINC00242-3. As shown in Fig. 2a, all three anti-LINC00242 constructs knock downed LINC00242 in HGC27 cells, whereas the si-LINC00242-3 knocked downed LINC00242 mostly. CCK-8 assay and Transwell chamber assays results revealed declined HGC27 cell viability, invasion, and migration upon LINC00242 knockdown (*p* < 0.05) (Fig. 2b–d). Western blot analysis showed decreased protein expression of N-cadherin, Vimentin, MMP-2 and MMP-9 in HGC27 cells upon LINC00242 knockdown (*p *< 0.05) (Fig. 2e). In addition, LINC00242 knockdown resulted in decreased tube formation of HBMVECs in HGC27 cell coculture (Fig. 2f). These data suggested that LINC00242 knockdown could inhibit GC cell migration, invasion, and in vitro angiogenesis.

### LINC00242 positively regulates FOXC1 expression by competitively binding to miR-141

**Fig. 3:**
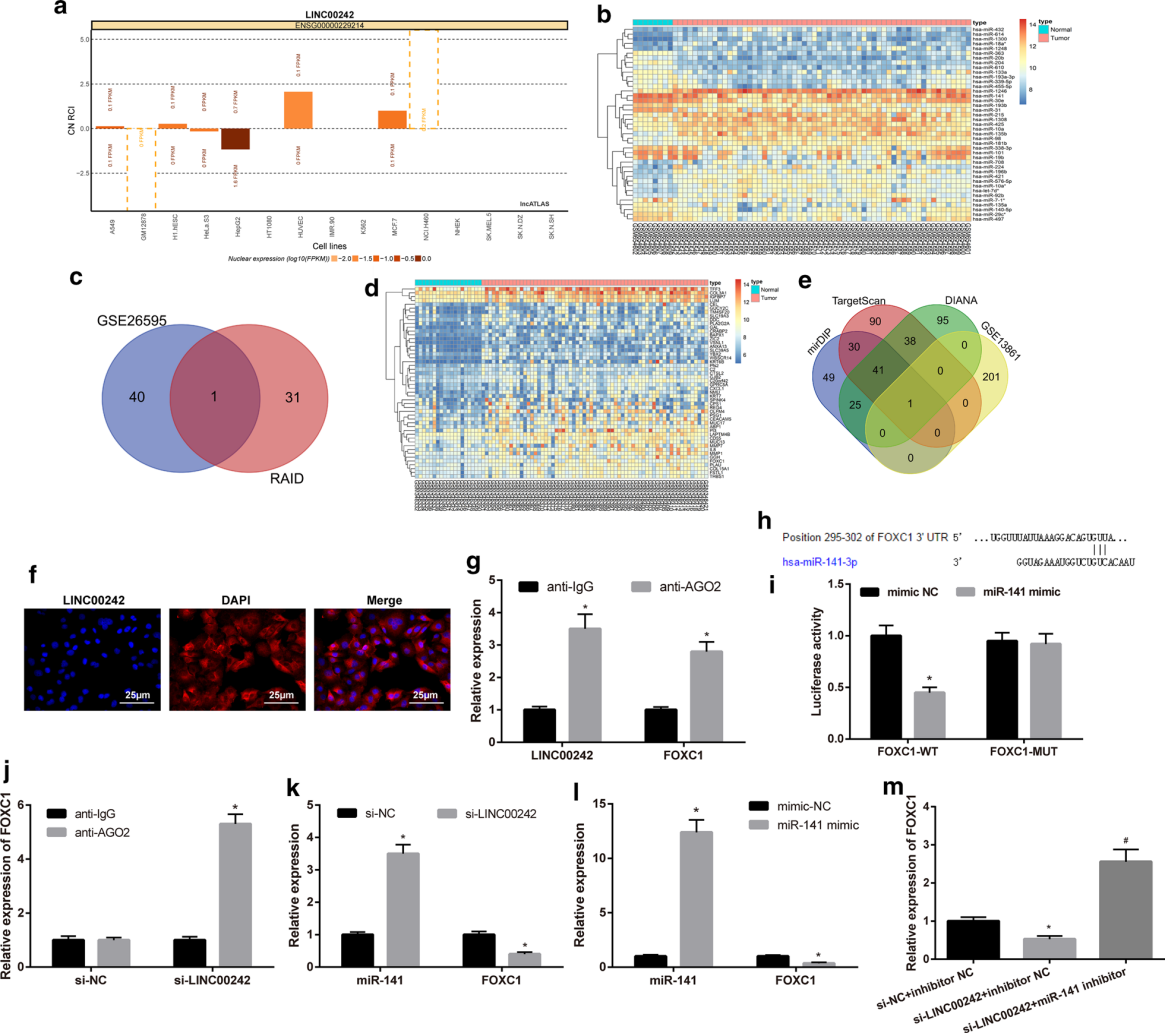
LINC00242 interacts with miR-141 and positively regulats FOXC1, a target gene of miR-141. **a** The predicted localization of LINC00242. The abscissa represents different cell lines and the ordinate represents the localization of LINC00242; the histogram above the 0 line represents the expression mainly in the cytoplasm, and the histogram below the 0 line represents the expression mainly in the nucleus. **b** A heat map illustrating differentially expressed miRNAs from GC-related GSE26595 dataset. **c** The intersecting miRNAs from the GSE26595 dataset and the RAID database (http://www.rna-society.org/raid/search.html). **d** A heat map illustrating some significantly upregulated genes from the GSE13861 dataset. **e** The intersecting genes from the mirDIP (http://ophid.utoronto.ca/mirDIP/index.jsp#r), TargetScan (http://www.targetscan.org/vert_71/) and DIANA (http://diana.imis.athena-innovation.gr/DianaTools/index.php?r=microT_CDS/index) databases and the GSE13861 dataset. **f** Subcellular localization of LINC00242 detected by FISH assay (× 400, scale bar = 25 μm). Green represents LINC00242 expression and blue represents the stained nucleus. **g** The binding of LINC00242 and FOXC1 to AGO2 assessed by RIP assay. * indicates *p *< 0.05 compared with IgG. **h** Putative miR-141 binding sites in the FOXC1 3′UTR by a web-available prediction (www.targetscan.org). **i** The luciferase activity at the promoter of the reporter gene containing FOXC1-WT and FOXC1-MUT upon miR-141 mimic transfection. * indicates *p *< 0.05 compared with mimic NC. **j** Expression of FOXC1 binding to using anti-AGO2 antibody in upon LINC00242 knockdown detected by RIP assay. * indicates *p *< 0.05 compared with si-NC. **k** Expression of miR-141 and FOXC1 in cells upon si-LINC00242 transfection detected by RT-qPCR, normalized to U6 and GAPDH respectively. * indicates *p *< 0.05 compared with si-NC. **l** Expression of FOXC1 in cells transfected with miR-141 mimic detected by RT-qPCR, normalized to GAPDH. * indicates *p *< 0.05 compared with mimic NC. **m** Expression of FOXC1 in cells transfected with si-LINC00242 or miR-141 inhibitor detected by RT-qPCR, normalized to GAPDH. Results are presented as mean ± S.D. of three technical replicates. * (compared with si-NC + inhibitor NC) and # (compared with si-LINC00242 + inhibitor NC) indicate *p *< 0.05 by unpaired *t*-test or one-way ANOVA followed by Tukey’s test

Next, we predicted the localization of LINC00242 and found that LINC00242 was expressed in both cytoplasm and nucleus, but mainly in cytoplasm (Fig. 3a). In order to further understand the mechanism of LINC00242 in GC, we retrieved GC-related GSE26595 dataset from the GEO database and found 41 significantly differentially expressed miRNAs (Fig. 3b). At the same time, 32 potential downstream miRNAs regulated by LINC00242 were obtained following prediction by the RAID database. Figure 3c revealed that there was one overlap miR-141 in the poorly expressed miRNAs from the GSE26595 dataset and the RAID database. We then analyzed another GSE13861 dataset retrieved from the GEO database and obtained 202 upregulated genes in GC (Fig. 3d). Concurrently, the downstream target genes of miR-141 were predicted by the mirDIP, TargetScan and DIANA databases. Then the first 200 genes predicted by the mirDIP (the score greater than 0.8), TargetScan and DIANA databases were selected and intersected with the downregulated genes from the GSE13861 dataset. The results shown in Fig. 3e revealed one overlapping FOXC1 gene. The above findings illustrated that LINC00242 is likely to regulate FOXC1 expression through miR-141, and ultimately affect the development of GC.

The FISH assay illustrated that LINC00242 was predominantly expressed in cytoplasm (Fig. 3f). The binding between miR-141 and LINC00242, miR-141 and FOXC1 detected by RIP assay is shown in Fig. 3g: LINC00242 and FOXC1 bound more to AGO2 antibody compared to IgG antibody, suggesting that miR-141 can specifically bind to LINC00242 and FOXC1. A biological prediction website (www.targetscan.org) revealed that miR-141 targeted and bound to FOXC1. The sequence of FOXC1 3′-UTR binding to miR-141 is illustrated in Fig. 3h. Dual-luciferase reporter gene assay results (Fig. 3i) showed that the luciferase activity of FOXC1-WT was reduced upon miR-141 mimic transfection (*p *< 0.05) while that of FOXC1-MUT showed no changes (*p* > 0.05). It was suggested that miR-141 could target FOXC1 and regulate its expression. In addition, RIP assay displayed that LINC00242 knockdown led to an increased expression of FOXC1 binding to AGO2 protein (Fig. 3j). RT-qPCR analysis showed a trend for increased miR-141 expression and decreased FOXC1 expression in HGC27 cells in response to si-LINC00242 transfection (*p *< 0.05) (Fig. 3k). Moreover, miR-141 expression was upregulated while FOXC1 expression was downregulated in HGC27 cells transfected with miR-141 mimic (*p *< 0.05) (Fig. 3l), which further confirmed the results shown in Fig. 3i: miR-141 targets FOXC1 and negatively regulates its expression. Additionally, FOXC1 expression was found to be reduced in HGC27 cells with si-LINC00242 + inhibitor NC (*p *< 0.05). However, si-LINC00242 + miR-141 inhibitor presented with an opposite trend in FOXC1 expression (*p *< 0.05) (Fig. 3m). These data collected suggested LINC00242 interacted with miR-141 and positively regulated FOXC1, a target gene of miR-141.

### LINC00242 knockdown retards HGC27 cell viability, migration and invasion, and tube formation of HBMVECs by regulating miR-141 and FOXC1

**Fig. 4:**
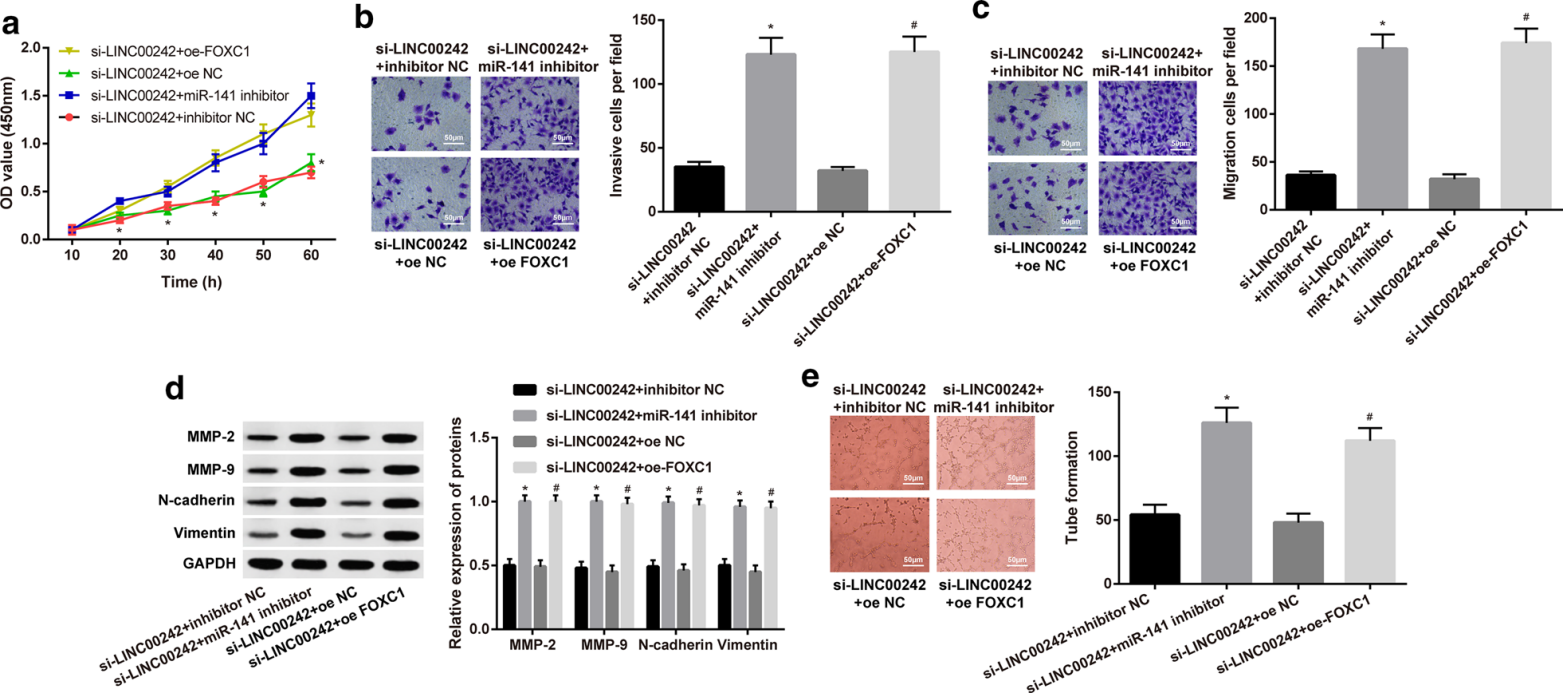
LINC00242 knockdown retards HGC27 cell viability, migration and invasion, and tube formation of HBMVECs by regulating miR-141 and FOXC1. HGC27 cells were treated with si-LINC00242 alone or with miR-141 inhibitor or oe-FOXC1. **a** HGC27 cell viability was measured by CCK-8 assay. **b** Representative view of HGC27 cells invading from Matrigel-coated chambers into lower ones and statistics of invading cells. **c** Representative view of HGC27 cells migrating from upper transwell chambers into lower ones and statistics of migrating cells. **d** Representative Western blots of MMP-2, MMP-9, N-cadherin, Vimentin and their quantitation analyses in HGC27 cells. **e***In vitro* tube formation of HBMVECs in HGC27 cells. Results are presented as mean ± S.D. of three technical replicates. * (compared with si-LINC00242 + inhibitor NC) and # (compared with si-LINC00242 + oe NC) indicate *p *< 0.05 by unpaired *t*-test (panel **b**–**e**) or repeated measurements ANOVA with Bonferroni corrections (panel **a**)

In order to further verify the effect of LINC00242 on the biological functions of GC by regulating FOXC1 and miR-141, we firstly detected HGC27 cell viability by CCK-8 assay. The results revealed that HGC27 cell viability was accelerated upon transfection of si-LINC00242 coupled with miR-141 inhibitor or oe-FOXC1, suggesting miR-141 inhibition or FOXC1 overexpression negated the effect of LINC00242 knockdown on HGC27 cell viability (*p* < 0.05) (Fig. 4a). Transwell chamber assays revealed inhibited HGC27 cell invasion and migration upon transfection of si-LINC00242 coupled with miR-141 inhibitor or oe-FOXC1, suggesting miR-141 inhibition or FOXC1 overexpression counteracted the effect of LINC00242 knockdown on HGC27 cell invasion and migration (Fig. 4b, c). Additionally, Western blot analysis demonstrated declined protein expression of MMP-2, MMP-9, N-cadherin, and Vimentin in HGC27 cells following transfection of si-LINC00242 coupled with miR-141 inhibitor or oe-FOXC1, suggesting miR-141 inhibition or FOXC1 overexpression abrogated the effect of LINC00242 knockdown on these proteins (*p* < 0.05) (Fig. 4d). The subsequent results from tube-forming test showed that transfection of si-LINC00242 + miR-141 inhibitor and si-LINC00242 + oe-FOXC1 resulted in enhanced tube formation of HBMVECs in HGC27 cell coculture (Fig. 4e). The aforementioned data revealed that LINC00242 knockdown could inhibit the viability, invasion, migration and tube formation of GC cells through promotion of miR-141 and inhibition of FOXC1.

### LINC00242 knockdown hampers the tumorigenesis of GC cells and angiogenesis in vivo

**Fig. 5:**
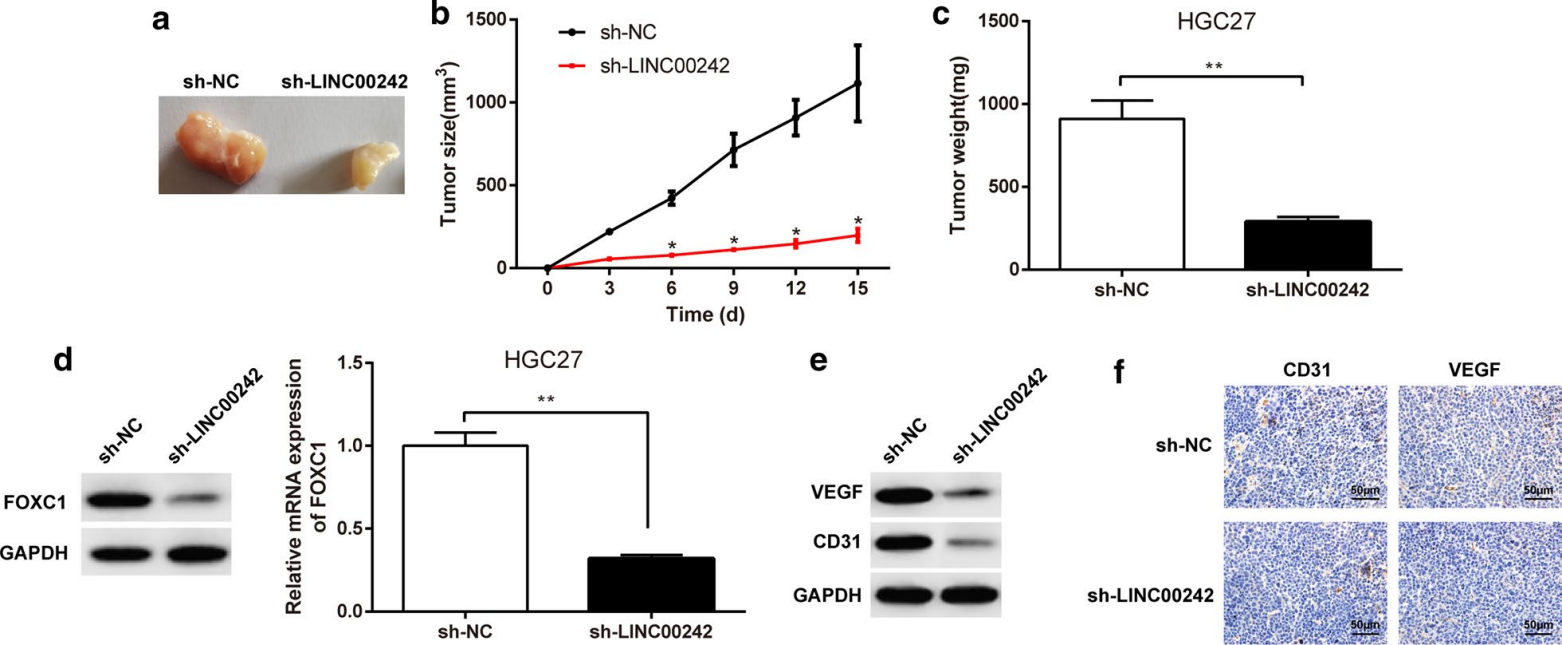
LINC00242 knockdown hampers the tumorigenesis of GC cells and angiogenesis in vivo. **a**–**c**, The volume and mass of GC xenografts by HGC27 cells upon LINC00242 knockdown. **d, e**, Representative Western blots of FOXC1, VEGF, CD31, and their quantitation analyses in tissues of GC xenografts by HGC27 cells upon LINC00242 knockdown. **f** Immunohistochemistry staining for VEGF and CD31 in tissues of GC xenografts by HGC27 cells upon LINC00242 knockdown (× 200, scale bar = 50 μm). Results are presented as mean ± S.D. of three technical replicates. * (compared with sh-NC) indicates *p *< 0.05 and ** (compared with sh-NC) indicates *p *< 0.01 by paired *t*-test

Since we demonstrated that LINC00242 knockdown could repress GC cell growth, migration, invasion, and angiogenesis in vitro, we continued to verify its tumor-suppressive role in GC in vivo. As shown in Fig. 5a, the size of GC xenografts was decreased upon LINC00242 knockdown. The growth of GC xenografts in mice was monitored every 3 days after implantation duration of 15 days, and we observed attenuated tumor growth upon LINC00242 knockdown (Fig. 5b). The weight of GC xenografts was likewise declined upon LINC00242 knockdown (Fig. 5c). These data indicated that LINC00242 knockdown can inhibit the tumorigenesis of GC cells in vivo. Subsequent western blot analysis results revealed that the protein expression of FOXC1, VEGF and CD31 was declined in tissues of GC xenografts upon LINC00242 knockdown (Fig. 5d, e). We likewise observed declined immunohistochemistry staining for VEGF and CD31 proteins in tissues of GC xenografts following LINC00242 knockdown (Fig. 5f). These results demonstrated that LINC00242 could promote the tumorigenesis of GC cells and angiogenesis in vivo.

## Discussion

**Fig. 6:**
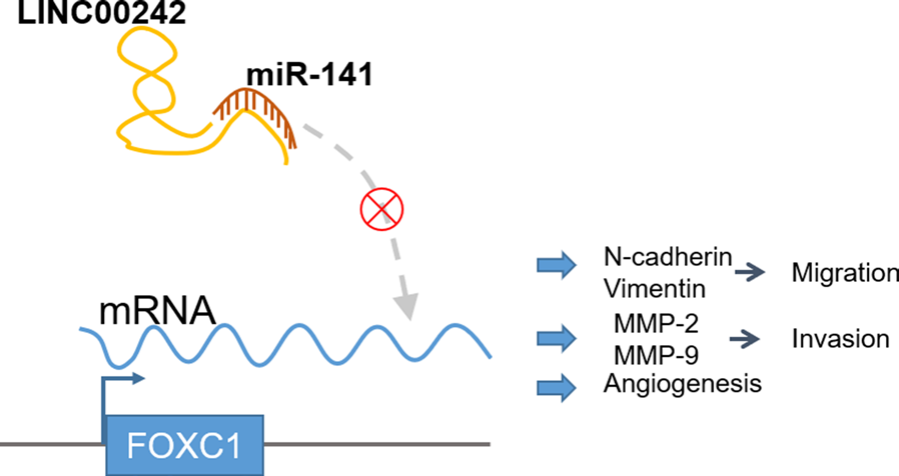
Mechanism diagram illustrating the regulatory network and function of LINC00242 in GC. LINC00242 is upregulated in GC tissues. Overexpressed LINC00242 competitively binds to miR-141 and consequently promotes FOXC1 expression, thus increasing expression of N-cadherin, Vimentin, MMP-2 and MMP-9, ultimately accelerating the tumorigenesis and angiogenesis in GC

Recently, the functions and mechanisms of lncRNAs have attracted increasing research attention either as tumor suppressors or oncogenes in prevalent cancers [15]. In the current investigation, the functional role of the LINC00242/miR-141/FOXC1 axis in the progression and metastasis of GC was explored. Collectively, our experimental data demonstrated that LINC00242 knockdown exerted anti-tumor effects in GC by inhibiting malignant cell growth, migration, invasion and angiogenesis through miR-141 target-inhibition of FOXC1 (Fig. 6).

Fundamentally, our results identified the high expression of LINC00242 in GC tissues and cells in association with dismal oncological outcomes of patients. To our knowledge, the expression pattern or clinical significance of LINC00242 in any type of cancers at present still remain less studied, yet there are reports on other dysregulated lncRNAs in GC. A novel lncRNA, HOXC-AS3, has been reported to be upregulated in GC tissues corresponding to advanced tumor node metastasis (TNM) stage and lower overall survival [16]. Likewise, increased expression of LINC00673 and LINC00978 in GC has been confirmed to exert oncogenic effects in association with tumor size and TNM stage [17, 18]. Importantly, lncRNAs are of great significance to the occurrence, development and metastasis of cancer by regulating a spectrum of biological processes functioning as oncogenes or tumor suppressors [19]. Therefore, subsequent gain- or loss-of-function assays were performed to elucidate the action of deregulated LINC00242 in GC.

Further attempts were made to unravel the effects of aberrantly expressed LINC00242 in GC by delivering si-LINC00242 into HGC27 cells. It was observed that depletion of LINC00242 weakened the growth, migration, invasion and angiogenesis potential of HGC27 cells as evidenced by diminished levels of N-cadherin, Vimentin, MMP-2, MMP-9, VEGF and CD31. The tumorigenicity of HGC27 cells was also suppressed in the presence of depleted LINC00242. Expression of N-cadherin, a biomarker of epithelial-mesenchymal transition, has been found to be elevated in GC samples in relation to lymph node metastasis, poor prognosis and advanced stage [20, 21]. The function of Vimentin in GC has been evaluated as a prognostic marker that positive expression of Vimentin has the capacity to foreshadow metastasis and dismal prognosis [22, 23]. Low expression of MMP-2 and MMP-9 are highly suggestive of better survival condition of patients with GC [24, 25]. Additionally, downregulation of MMP-2 and MMP-9 has been elaborated to involve in the antitumor activity of lysyl oxidase and secretory leukocyte protease inhibitor in GC [26, 27]. Moreover, VEGF has been deciphered to play a part in the functional role of MMP-2 in GC [28]. Angiogenesis is of great therapeutic significance to GC [4]. As a major regulator of angiogenesis, VEGF is highly expressed in GC, facilitating the malignancies of tumor cells and growth and metastasis of tumors [29]. In addition to VEGF, CD31 expression is also elevated to be implicated in tumor angiogenesis of gastrointestinal stromal tumors that elevated CD31 expression is correlated with worse prognosis [30]. Similar regulatory mechanism with regard to lncRNA MNX1 antisense RNA 1 (MNX1-AS1) has been unraveled that depleted lncRNA MNX1-AS1 can reduce expression of Vimentin and MMP-9, supporting its oncologic role in GC [31].

Additionally, the possible underlying regulatory mechanism was studied and results revealed that LINC00242 worked in tandem with miR-141 and FOXC1 in GC. To be specific, LINC00242 could bind to miR-141 to mediate the expression of FOXC1, a target gene of miR-141. Interaction between lncRNAs and miRNAs has been recognized to harbor potential function in tumorigenesis, including GC [32–34]. Largely in agreement with our finding, LINC01234 has been indicated to serve as an oncogene in the progression of GC by modulating the miR-204-5p/core-binding factor β axis [35]. Upregulation of LINC01296 has been demonstrated to exert growth-promoting effects in GC through interaction with miR-122 via MMP-9 [36]. Besides, miR-141 inhibition or overexpressed FOXC1 was shown to counterweigh the anti-tumor effects of LINC00242 knockdown in GC. By interacting with another lncRNA maternally expressed 3, miR-141 inhibition can offset its regulation on GC cell growth [37]. Also, overexpressed FOXC1 may be useful in predicting undesirable postoperative outcomes of patients with GC [38]. Notably, due to the limited time and funding, we were not allowed to investigate the exact expression patterns and functional roles of miR-141 and FOXC1 playing in GC, respectively, which requires further thorough research in the future for clinical rationale of the reported axis.

## Conclusion

To conclude, these findings in the present investigation shed light on the carcinogenic action of LINC00242 in GC through its interaction with miR-141 and FOXC1, providing a novel therapeutic candidate to develop treatment modalities against GC. However, standard operation procedures are still expected for analytical validation and clinical utility of lncRNAs as promising biomarkers from different perspectives.

## Data Availability

All data and materials are fully available without restriction. The data generated or analyzed during this study are included in this published article.

## Abbreviations

LncRNAs: Long non-coding RNAs
miRNAs: microRNAs
GC: Gastric cancer
FOXC1: Forkhead box C1
RIP: RNA immunoprecipitation
HBMVECs: Human brain microvascular endothelial cells
FBS: Fetal bovine serum
RT-qPCR: Reverse transcription quantitative polymerase chain reaction
IgG: Immunoglobulin G
UTR: Untranslated region
FISH: Fluorescence in situ hybridization
ANOVA: One-way analysis of variance
